# Comparison of Pancreatic Damage in Rats for Two Methods of Paraquat Administration

**DOI:** 10.3389/fphar.2021.611433

**Published:** 2021-04-20

**Authors:** Yanxia Gao, Linlin Hou, Yibo Wang, Yan Zhang, Shoutao Zhang, Yi Li, Yanan Jiang, Changju Zhu, Tongwen Sun, Guoyu Duan, Ding Yuan

**Affiliations:** ^1^ Emergency Department, The First Affiliated Hospital of Zhengzhou University, Zhengzhou, China; ^2^ Henan Key Laboratory of Bioactive Macromolecules, School of Life Sciences, Zhengzhou, China; ^3^ Emergency Department, Chinese Academy of Medical Sciences, Peking Union Medical College Hospital, Beijing, China; ^4^ Department of Pathophysiology, School of Basic Medical Sciences, Zhengzhou University, Zhengzhou, China

**Keywords:** paraquat poisoning, pancreatic damage, multiple organ damage, intragastric infusion, intraperitoneal injection

## Abstract

It is noted that elevated serum amylase levels suggesting pancreatic damage has an association with prognosis in PQ patients. This study aimed to determine whether PQ can cause pancreatic damage. The two conventional models (intragastric infusion (iG) and intraperitoneal injection (iP)) may exhibit different effects on the pancreas depending on whether or not they pass through the digestive tract. In this study, the rats were divided into four groups: the intragastric infusion group (PQ-iG, *n* = 45), intraperitoneal injection group (PQ-iP, *n* = 53), normal control group 1 (NC-iG, *n* = 6) and normal control group 2 (NC-iP, *n* = 6). Pancreatic damage was compared between groups using serum amylase activity assay, hematoxylin and eosin (H&E) staining, TUNEL assay, and transmission electron microscopy (TEM). Serum amylase levels in group PQ-iG were significantly higher than in group PQ-iP (*p* < 0.05). Examination of the H&E sections showed damage to the pancreas. Both experimental groups were displayed inflammatory infiltration within 9 h of PQ treatment. After 9 h, patchy necrosis was observed in group PQ-iP, when inflammatory infiltration was still the dominant pathology. Necrosis appeared and gradually worsened in group PQ-iG, in which necrosis was the dominant pathology. The TUNEL assay showed significantly higher numbers of apoptotic cells in the pancreas of PQ-groups than in the control NC- groups (*p* < 0.05). TEM showed expansive endoplasmic reticulum lumens and mitochondria swelling in the pancreas of the PQ-groups. It is concluded that both methods of modeling could cause pancreatic damage and the type and degree of damage would change over time. Note that pancreatic damage in group PQ-iG was more severe than that in group PQ-iP. Therefore, clinical practitioners should pay close attention to pancreatic damage caused by PQ, especially when the route of PQ administration was oral.

## Introduction

Paraquat (PQ) ingestion can lead to multiple organ failure (Melchiorri et al., 1995; Adachi et al., 2000; Zhang X. et al., 2019; Liu et al., 2019; Mirzaee et al., 2019). There is no specific antidote for PQ poisoning, and even small doses of PQ can cause death; the fatality rate of PQ is as high as 50–80% (Zhang L. et al., 2019; Zhang X. et al., 2019; Wu et al., 2019). The cause of PQ poisoning deaths is usually recorded as acute respiratory failure (Wu et al., 2019; Gao F. et al., 2020). Some clinical studies have shown elevated serum amylase levels in PQ poisoning patients, which suggests that PQ may cause pancreatic damage (Soontornniyomkij and Bunyaratvej, 1992; Wang and Qian, 2005; Gong et al., 2016). It has been shown that patients diagnosed with PQ poisoning who have elevated levels of serum amylase have a worse prognosis than other patients (Gil et al., 2009; Li et al., 2015; Huang et al., 2020).

In clinical patients, PQ is usually taken orally. However, in experimental studies, PQ is often administered by intraperitoneal injection (iP) because it is easy to use and the dose is controllable (Wunnapuk et al., 2013; Chen et al., 2015; Zhang et al., 2018). The biggest difference between iP and iG is in passage through the digestive tract. The pancreas is a digestive organ that is anatomically close to the digestive tract. In our previous animal model study, in which PQ was administered to rats by intragastric infusion (iG), we found that PQ poisoning increased serum amylase levels in the rats; histopathological examination of rat pancreas showed that the pancreas presented inflammatory cell infiltration and cell necrosis (Gao Y. et al., 2020). Regarding pancreatic damage, there is still a controversy in academia. Some researchers believe that PQ poisoning-induced increased amylase may be caused by gastrointestinal damage, which is not strong evidence that pancreatic damage exists (Huang et al., 2020). However, others maintain that PQ in the digestive tract may retrograde into the pancreatic duct due to causes like vomiting and gastric lavage after PQ poisoning, leading to pancreatic damage (Gong et al., 2016). Further investigation of whether PQ poisoning by iP leads to pancreatic damage and differences in the progression of PQ poisoning between administration by iG and iP is merited.

In this study, we observed and compared pancreatic damage caused by PQ when administered by iG and iP, which had not been studied before. The results showed that PQ could cause pancreatic damage when it was either administered by iG or iP; the type and degree of damage would change over time, and there were differences between the two groups. Thus, clinical practitioners are recommended to pay attention to pancreatic damage caused by PQ.

## Materials and Methods

### Animal Protocol

Healthy male Sprague-Dawley (SD) rats, purchased from Beijing Vital River Laboratory Animal Technology Co., Ltd., weighing 180–220 g, were used in this study. The rats were fed under a 12 h light/12 h dark cycle with unlimited food and water for one week prior to the experiment. Ethical approval for this study was obtained from the Animal Ethics Committee of Zhengzhou University in accordance with institutional guidelines for the care and use of animals for scientific purposes (permit number 2019-KY-191).

A total of 110 rats were divided into four groups: normal control group 1 (NC-iG, *n* = 6), normal control group 2 (NC-iP, *n* = 6), intragastric infusion group (PQ-iG, *n* = 45) and intraperitoneal injection group (PQ-iP, *n* = 53). Rats in group NC-iG received 1 ml 0.9% normal saline by iG. Rats in NC-iP group received 1 ml 0.9% normal saline by iP. PQ powder (Sigma-Aldrich, United States) was suspended and dissolved in 0.9% normal saline. The PQ-iG group was administered PQ by iG infusion at a dose of 120 mg/kg in a total volume of 1 ml 0.9% normal saline, and the PQ-iP group was administered PQ by iP injection at a dose of 35 mg/kg in a total volume of 1 ml 0.9% normal saline. The dose was determined with reference to the half lethal dose of PQ poisoning induced by iG and iP in rats (Dinis-Oliveira et al., 2008). The rats were deeply anesthetized by intraperitoneal injection of 50 mg/kg pentobarbital sodium and sacrificed randomly at 3, 6, 9, 12, 24, 48 and 72 h after PQ administration, at least three rats were randomly selected from each group at each time point. The serum was isolated and stored in a −80°C refrigerator for later use; pancreas were harvested and soaked in 4% paraformaldehyde.

### Amylase Activity Assay

Levels of rat serum amylase were measured using an amylase activity assay kit (Cat.MAK009, Sigma-Aldrich, United States). The experimental procedure strictly followed the kit manufacturer’s instructions.

### Hematoxylin and Eosin (H&E) staining

Rat tissues were fixed in 4% paraformaldehyde and embedded in paraffin. The specimens were then sectioned at a thickness of 5 μm, dewaxed using xylene, dehydrated using an alcohol gradient, and stained with H&E. The sections were counterstained with hematoxylin and mounted. The stained sections were examined using an optical microscope (Leica, Germany). The scoring method described by Schmidt et al. was used to evaluate pancreatic histopathology in the rat tissue (Schmidt et al., 1992).

### TUNEL Assay

Apoptosis of pancreatic acinar cells was investigated using a TUNEL apoptosis assay (Promega, United States) according to the manufacturer’s instructions. The section was dewaxed with xylene and dehydrated with alcohol with a reduced concentration gradient. After dehydration, tissue sections were digested with 2% protease K, incubated at 37°C for 20 min, and then washed 3 times with phosphate buffer saline (PBS). Each section was drizzled with 40 µL TDT (1:10) and incubated for 120 min at 37°C. After washing 3 times with PBS, the section was stained with 50 µL DAPI (1:100), incubated at 37°C for 2 min, and then soaked in PBS 3 times, for 5 min each time. The slices were all sealed with an anti-fluorescence quenching agent, and images were collected under a fluorescence microscope (Leica).

### Transmission Electron Microscopy

The rat pancreas were imaged using transmission electron microscopy (TEM) to observe mitochondria and endoplasmic reticulum morphology. Pancreatic tissue from all experimental groups was immediately fixed in 2.5% glutaraldehyde (Solarbio, Beijing, China). The dissected pancreatic tissue was washed with PBS and post-fixed in 2% osmium tetroxide for 2 h at room temperature. Fixed tissue was dehydrated in a series of graded alcohols and embedded in Epon resin. The resin was polymerized, and blocks were sectioned on a microtome (Leica). The sections were double-stained with uranyl acetate and lead citrate and then examined and imaged with a transmission electron microscope (Leica).

### Statistical Analysis

SPSS software version 21.0 (SPSS Inc., United States) was used for statistical analysis. The Kolmogorov–Smirnov test was used to assess the normality of the distribution. The measurement data conforming to the normal distribution were described as mean plus or minus standard deviation (mean ± SD). Samples from two groups were compared using independent sample *t*-tests and correlation analysis, and samples from four groups were compared using one-way analysis of variance (ANOVA), followed by the least significant difference (LSD) test. Measurement data that did not conform to the normal distribution were described by the median and interquartile range [M(p25, p75)]. The Wilcoxon rank-sum test was used for comparison between two groups, and the Kruskal–Wallis rank-sum test was used for comparisons between three or four groups, followed by the Bonferroni test. Differences were considered significant at *p* < 0.05.

## Results

### Rat Mortality

**FIGURE 1:**
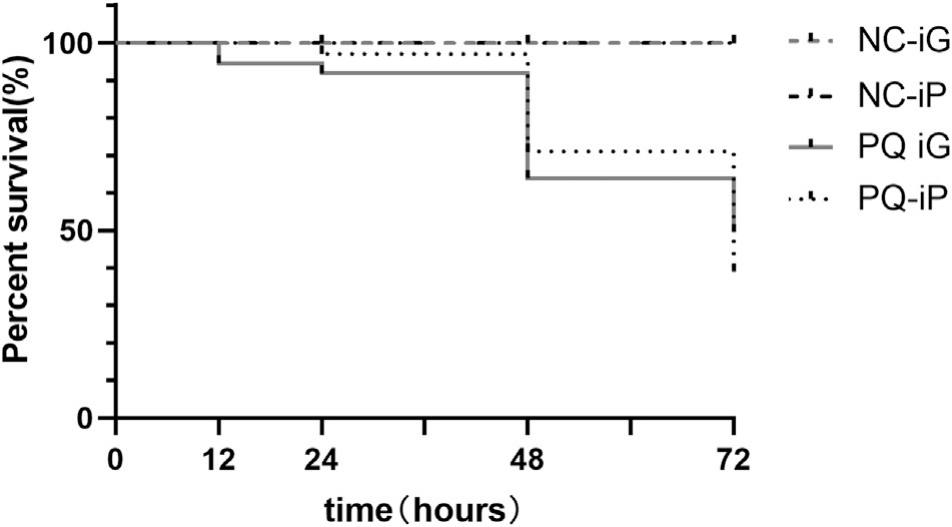
Survival curves of rats in all groups after PQ administration.

During the experiment, we recorded the number of animals in the four groups that had died at each time point and drew a cumulative survival curve (Figure 1). Rats in the NC- groups survived until they were sacrificed at the end of the experiment. Figure 1 shows cumulative survival after exposure to PQ. A sharp increase in mortality was observed at 48 h after PQ administration. At 48 h, the cumulative mortality rates were 40% in group PQ-iG and 29% in group PQ-iP. At 72 h, the two groups had similar cumulative mortality rates: the cumulative mortality rates were 56% in group PQ-iG and 61% in group PQ-iP.

### Pathological Changes in Pancreas and Serum Amylase Activity

**FIGURE 2:**
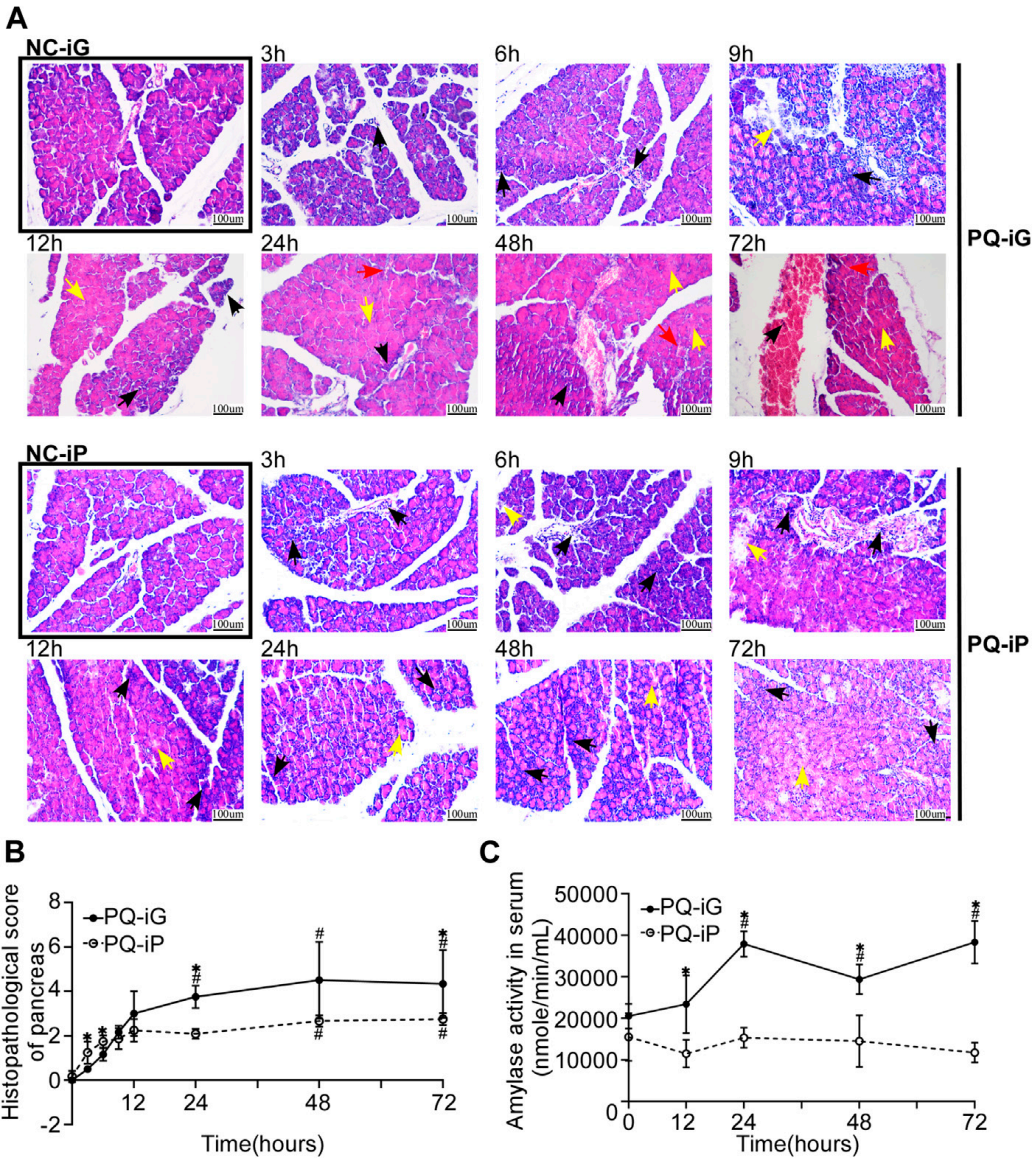
Changes in the pancreas due to iG and iP administration of PQ. **(A)** Typical pathological changes in H&E stained pancreatic tissue sections in groups PQ-iG and PQ-iP at various times of observation (magnification ×200; scale bar: 100 μm; black arrow = inflammatory cell infiltration; yellow arrow = necrotic cell; red arrow = hemorrhage); **(B)** Pathological damage scores of rat pancreas in groups PQ-iG and PQ-iP at each observation time point; **(C)** Serum amylase activity ( nmole/ min/ ml) of rats in groups PQ-iG and PQ-iP. The corresponding NC- groups values were taken for 0 h after PQ treatment. ^*^ *p* < 0.05: the difference between the PQ-iG and PQ-iP groups was statistically significant at this time point; ^#^ *p* < 0.05: the difference was statistically significant in comparison with the corresponding NC group. PQ paraquat; iG: intragastric infusion; iP: intraperitoneal injection.

Serum amylase levels were measured (Figure 2C). Amylase activity for rats in group PQ-iG was higher than in group NC-iG; the difference was statistically significant (*p* < 0.05) after 24 h. Amylase activity did not significantly increase in group PQ-iP over group NC-iP (*p* = 0.12). There was no statistically significant difference between the two NC- groups (*p* = 0.086). Group PQ-iG showed higher levels than group PQ-iP, and the difference was statistically significant (*p* < 0.05).

The pancreas tissue was stained with H&E at each observation time point to enable us to observe and compare changes in pancreatic damage between the two methods of PQ administration (iG and iP). Figure 2A shows that there was no obvious pathological damage in the NC- groups; the pancreatic lobule was intact, the demarcation was clear, and there was almost no inflammatory cell infiltration, necrosis, or hemorrhage. In group PQ-iG, inflammatory cell infiltration had occurred at 3 h, acinar cell fusion and necrosis had occurred at 9 h, and vascular rupture and hemorrhage had occurred at 24 h. In group PQ-iP, inflammatory cell infiltration had occurred at 3 h, and acinar cell fusion and necrosis had occurred at 6 h. Before 9 h, inflammatory infiltration was the predominant presentation in both groups. After 9 h, patchy necrosis was observed in group PQ-iP, in which inflammatory infiltration was still predominant. Necrosis appeared and gradually increased in group PQ-iG to become predominant.

We allocated pathological damage scores according to the scoring method given by Schmidt et al. (Schmidt et al., 1992) to quantify the pancreatic damage (Figure 2B). There was no difference in pathological damage scores between the NC- groups (*p* = 0.174). The pathological damage scores of the PQ-groups were higher than those of the corresponding NC- groups. In group PQ-iG, the difference in scores at 24 h, when compared with the group NC-iG, was statistically significant (*p* < 0.05). In group PQ-iP, the difference in scores at 48 h, when compared with the group NC-iP, was statistically significant (*p* < 0.05). At 3 and 6 h, the score of group PQ-iP was higher than that of group PQ-iG; at 9 h and thereafter, the score of group PQ-iG was higher than that of group PQ-iP. The scores for the NC- groups at 0 h were taken as the baselines. These results suggest PQ administration by both iG and iP causes pancreatic damage. The initial pancreatic damage in group PQ-iP was more severe than in group PQ-iG but pancreatic damage at 72 h was less severe than in group PQ-iG.

### Effects of PQ Poisoning on Pancreatic Tissue Apoptosis

**FIGURE 3:**
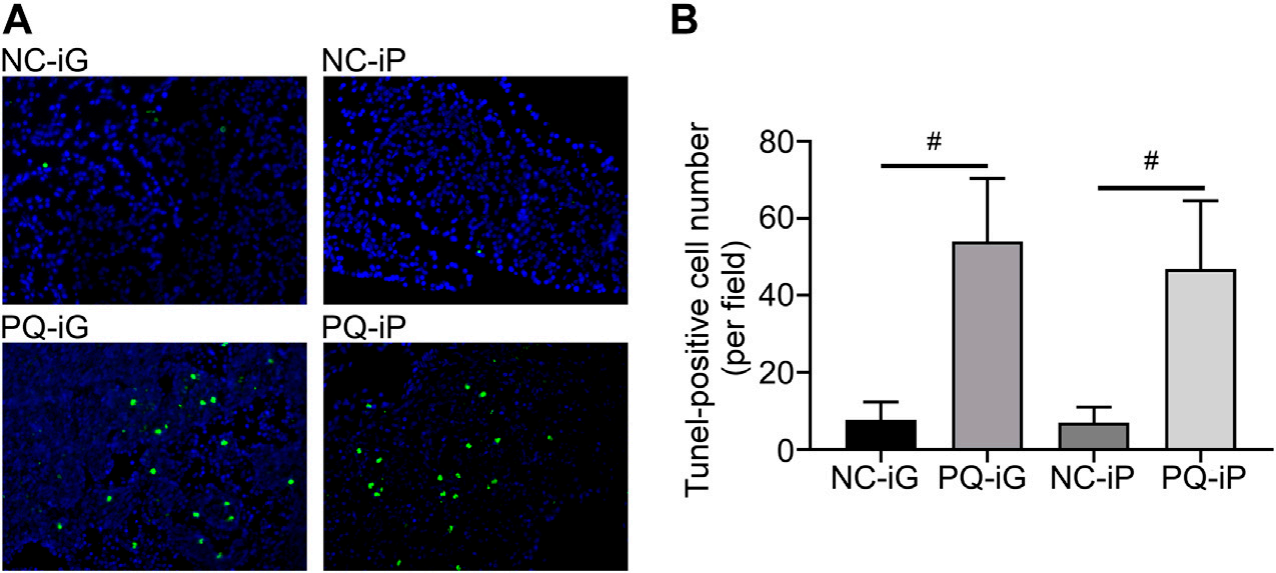
TUNEL staining assay was used to determine apoptosis in pancreatic tissue. **(A)** Typical TUNEL assay results for pancreatic tissue in each group (magnification ×200); **(B)** TUNEL stained apoptotic cell numbers. ^#^ *p* < 0.05: the difference was statistically significant when compared to the corresponding NC- group.

Apoptosis is a hallmark of pancreatitis (Wang et al., 2019; Zhou et al., 2019). Figure 2B showed that the pathological damage scores of PQ-groups stabilized at 48 h. Thus we selected pancreatic wax blocks from 48 h after PQ administration for TUNEL assay to quantify the degree of apoptosis. In Figure 3A, green indicates the apoptotic cells and increased green fluorescence indicates increased cell apoptosis in tissue. The number of TUNEL-positive cells for each group was calculated by ImageJ (Figure 3B); there was no difference between the NC-iG and NC-iP groups. Significantly higher TUNEL-positive cell numbers were found in the PQ-groups than in the corresponding NC- groups (*p* < 0.05). The TUNEL-positive cell count for group PQ-iG was higher than for group PQ-iP; there was no statistically significant difference between the two groups (*p* = 0.4589).

### Changes in Pancreatic Organelles

**FIGURE 4:**
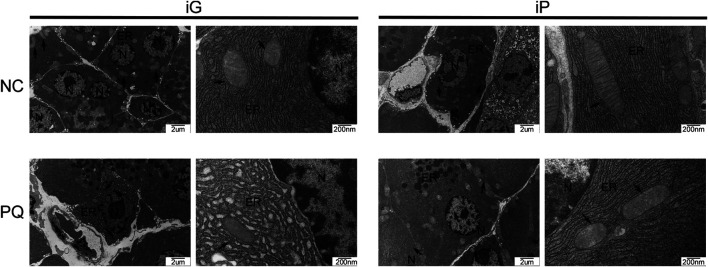
Typical TEM photos of pancreatic tissue sections (bar = 2 μm or 200 nm); N: nucleus, mt: mitochondria, black arrow: mitochondria, ER: endoplasmic reticulum, TEM: transmission electron microscopy, NC: normal control, PQ: paraquat poisoning, iG: intragastric infusion, iP: intraperitoneal injection.

TEM was used to examine changes in the ultrastructure, particularly cellular organelles, of pancreatic tissue 48 h after exposure to PQ. Pancreas sections in the NC- groups had regular structures in the nuclei, with evenly distributed endoplasmic reticulum and normal mitochondria that were intact. In the PQ-groups, endoplasmic reticulum lumen and endoplasmic reticulum disorder were observed, the endoplasmic reticulum showed mild to moderate diffuse endoplasmic reticulum lumen dilation in PQ-iG groups, and mild local endoplasmic reticulum lumen dilation in PQ-iP groups. Mitochondria showed mild local edema in PQ-iG groups and in PQ-iP groups (Figure 4).

## Discussion

In this study, we have focused on pancreatic damage due to PQ poisoning in two conventional rat models. The main findings of our study are: 1. PQ could cause pancreatic damage when it was administered either by iG or iP. Pancreatic damage in group PQ-iG was more severe than that in group PQ-iP; 2. the type and degree of damage would change over time, and there were differences between the two groups.

Some previous studies had reported the abnormal pancreatic enzymes, but few studies have focused on pancreas (Soontornniyomkij and Bunyaratvej, 1992; Wang and Qian, 2005; Li et al., 2015; Gong et al., 2016). There are only two previous basic studies of pancreatic damage due to PQ poisoning. One is our previous study (Gao Y. et al., 2020), a rat model of PQ poisoning by iG administration. The other is the report of an experiment by Silfeler et al. (Silfeler et al., 2015), who induced PQ poisoning in rats by iP administration. In this study, we found that serum amylase levels increased significantly in group PQ-iG but not in group PQ-iP compared with the corresponding NC- groups. PQ poisoning by iG administration led to elevated levels of serum amylase, which is consistent with our previous research (Gao Y. et al., 2020) and clinical reports (Soontornniyomkij and Bunyaratvej, 1992; Wang and Qian, 2005; Gong et al., 2016). Serum amylase levels did not significantly increase in group PQ-iP compared with group NC-iP, a finding that is consistent with that of Silfeler et al. The fact that the increased serum amylase levels in group PQ-iG is more significant than that in group PQ-iP can be explained as follows: 1. pancreatic damage in group PQ-iG was more severe than in group PQ-iP; 2. the increased serum amylase levels in group PQ-iG may have been caused in part by gastrointestinal damage (Liu et al., 2016; Huang et al., 2020).

Silfeler et al. found inflammatory cell infiltration, edema, and congestion in pancreatic tissue. We found in this study that inflammatory infiltration was predominant in both groups within 9 h of PQ administration. After 9 h, patchy necrosis was observed in group PQ-iP, in which inflammatory infiltration was still predominant; necrosis appeared and gradually increased in group PQ-iG to become predominant. The pathological damage score for group PQ-iP was higher than for group PQ-iG at 9 h after PQ administration; after 9 h, the score for group PQ-iG was higher than for group PQ-iP. The type and degree of damage changed over time, and there were differences between the two groups. The reason the score for group PQ-iP was higher than for group PQ-iG may be that PQ was absorbed directly within the peritoneal cavity in rats in group PQ-iP but absorbed into the blood through the digestive tract in rats in group PQ-iG. The reason pancreatic damage in group PQ-iG was more severe than in group PQ-iP may be that there are two pathways for pancreatic damage to occur in group PQ-iG: through corrosion of gastrointestinal tract and microecology (Cen et al., 2018), and by inflammatory reaction and oxidative stress in response to PQ absorbed in the bloodstream. PQ administered by iP causes pancreatic damage only through inflammatory reaction and oxidative stress due to PQ absorbed into the bloodstream. We thus suggest that the animal model method made by iG is much closer to the pancreatic damage caused by PQ poisoning in the clinical study.

PQ causes cell apoptosis in lungs (Seo et al., 2014; Cui et al., 2019; Zhang et al., 2020), kidneys (Hu et al., 2019), liver (El-Boghdady et al., 2017; Qian et al., 2019) and nerves (Fujimori et al., 2012; Ju et al., 2019). There have been no studies into whether PQ causes apoptosis in pancreatic cells. We observed in this study that PQ causes pancreatic cell apoptosis. The TUNEL assay results show higher apoptotic cell numbers were found in PQ-groups in contrast with the corresponding NC- groups.

The pancreas is a digestive organ that synthesizes many digestive enzymes. Pancreatic acinar cells are rich in endoplasmic reticulum and mitochondria (Habtezion et al., 2019). Dysfunction of the endoplasmic reticulum and mitochondria indicates impaired pancreatic function. We also observed, through TEM, changes in pancreatic organelles caused by PQ. The results of TEM showed that the endoplasmic reticulum showed mild to moderate diffuse endoplasmic reticulum lumen dilation in PQ-iG groups, and mild local endoplasmic reticulum lumen dilation in PQ-iP groups. Mitochondria showed mild local edema in PQ-iG groups and in PQ-iP groups. TEM also confirmed that PQ can cause pancreatic damage.

## Conclusion

In conclusion, PQ could cause pancreatic damage and its type and degree would change over time. Pancreatic damage in group PQ-iG was more severe than that in group PQ-iP. Clinical practitioners should focus on pancreatic damage caused by PQ, especially when the route of PQ administration was oral.

## Data Availability

The raw data supporting the conclusions of this article will be made available by the authors, without undue reservation, to any qualified researcher.
